# Decoding human in vitro terminal erythropoiesis originating from umbilical cord blood mononuclear cells and pluripotent stem cells

**DOI:** 10.1111/cpr.13614

**Published:** 2024-03-18

**Authors:** Xiaoling Wang, Wei Zhang, Siqi Zhao, Hao Yan, Zijuan Xin, Tiantian Cui, Ruge Zang, Lingping Zhao, Haiyang Wang, Junnian Zhou, Xuan Li, Wen Yue, Jiafei Xi, Zhaojun Zhang, Xiangdong Fang, Xuetao Pei

**Affiliations:** ^1^ Stem Cell and Regenerative Medicine Lab Beijing Institute of Radiation Medicine Beijing PR China; ^2^ Beijing Institute of Genomics & China National Center for Bioinformation Chinese Academy of Sciences Beijing PR China; ^3^ Institute for Stem Cell and Regeneration Chinese Academy of Sciences Beijing PR China; ^4^ Sino‐Danish College University of Chinese Academy of Sciences Beijing PR China; ^5^ Beijing Key Laboratory of Genome and Precision Medicine Technologies Beijing PR China; ^6^ College of Life Sciences University of Chinese Academy of Sciences Beijing PR China; ^7^ Savaid Medical School University of Chinese Academy of Sciences Beijing PR China; ^8^ School of Future Technology University of Chinese Academy of Sciences Beijing PR China

## Abstract

Ex vivo red blood cell (RBC) production generates unsatisfactory erythroid cells. A deep exploration into terminally differentiated cells is required to understand the impairments for RBC generation and the underlying mechanisms. Here, we mapped an atlas of terminally differentiated cells from umbilical cord blood mononuclear cells (UCBMN) and pluripotent stem cells (PSC) and observed their dynamic regulation of erythropoiesis at single‐cell resolution. Interestingly, we detected a few progenitor cells and non‐erythroid cells from both origins. In PSC‐derived erythropoiesis (PSCE), the expression of haemoglobin switch regulators (BCL11A and ZBTB7A) were significantly absent, which could be the restraint for its adult globin expression. We also found that PSCE were less active in stress erythropoiesis than in UCBMN‐derived erythropoiesis (UCBE), and explored an agonist of stress erythropoiesis gene, TRIB3, could enhance the expression of adult globin in PSCE. Compared with UCBE, there was a lower expression of epigenetic‐related proteins (e.g., CASPASE 3 and UBE2O) and transcription factors (e.g., FOXO3 and TAL1) in PSCE, which might restrict PSCE's enucleation. Moreover, we characterized a subpopulation with high proliferation capacity marked by CD99^high^ in colony‐forming unit‐erythroid cells. Inhibition of CD99 reduced the proliferation of PSC‐derived cells and facilitated erythroid maturation. Furthermore, CD99–CD99 mediated the interaction between macrophages and erythroid cells, illustrating a mechanism by which macrophages participate in erythropoiesis. This study provided a reference for improving ex vivo RBC generation.

## Graphical abstract

scRNA‐seq and cell typing of late‐stage umbilical cord blood mononuclear cells‐ and pluripotent stem cell (PSC)‐derived cells were conducted. Stress erythropoiesis was more active in UCBMN‐derived erythropoiesis (UCB‐E) than in PSC‐derived erythropoiesis (PSC‐E). Regulators that impair adult globin expression and enucleation between UCB‐E and PSC‐E were compared at single cell level. CD99^high^ progenitor cells were a proliferating colony forming unit erythroid subpopulation.

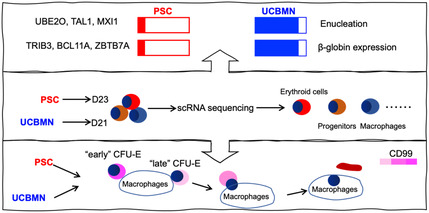

## 1 INTRODUCTION

Immortalized cell lines, such as pluripotent stem cells (PSCs), are ideal, stable, and a theoretically infinite source of red blood cell (RBC) generation in vitro. However, PSC‐generated erythrocytes have also shown the weakness of extremely low‐enucleation ratios and insufficient β‐globin expression.^1^, ^2^ Since the multi‐lineage differentiation potential, umbilical cord blood (UCB) mononuclear cells (UCBMNs) have become one of the most valuable primary cell sources for RBC generation. Compared with PSCs, UCBMN‐derived erythroid progenitors possess higher proliferation capacities and their offspring RBCs have shown higher enucleation ratios and β‐globin expression levels.^3^ Recently, comparative bulk RNA‐seq analysis between embryonic stem cell and UCB‐CD34^+^‐derived erythroid cells provided insights into the limited expansion and defective enucleation of embryonic stem cell‐origin RBCs at a “development stage” resolution,^4^ but this study was limited in analysing the cell composition, pseudo‐time trajectories and cell–cell communications of the regenerated cells from both origins. Deciphering the ex vivo erythroid cell generation at single cell resolution is urgently required to explore the mechanisms underlying ex vivo erythropoiesis limitations that have been largely unknown.

Due to the high‐resolution genomic information, single‐cell sequencing technology strongly improves our understanding of erythropoiesis and the contribution of each cell type within the cell population.^5^ A previous study constructed a haematopoietic development model characterized by cell population heterogeneity at early stages of erythropoiesis using single‐cell sequencing technology.^6^ Xin et al. determined specific regulatory networks that control ex vivo induced PSC‐derived erythropoiesis in embryoid body culture, providing critical clues toward improvement of the process, but this study was limited in that it focused on very early developmental erythropoietic stages. A previous study assessed the transcription dynamics of terminally differentiated human erythroblasts from human cord blood and bone marrow cells at single cell resolutions^7^; this study specifically focused on the mechanisms of in vivo terminal erythropoiesis in orthochromatic erythroblasts (Ortho‐E) from the two primary sources. Currently, single‐cell transcriptomic differences between UCBMN‐derived (UCBE) and PSC‐derived erythroid (PSCE) at terminal stages and their underlying regulatory mechanism remain unknown.

In this study, we comprehensively mapped and compared the atlases of ex vivo UCBMN‐ and PSC‐derived terminally differentiated cells and explored the mechanisms underlying limitations associated with ex vivo RBC production, particularly low adult globin expression and erythroid enucleation. We characterized the major components, colony‐forming unit‐erythroid (CFU‐E) and macrophages, in the terminal populations and provided new clues for facilitating ex vivo erythropoiesis. This study aimed to decipher ex vivo terminal erythropoiesis at the single cell level and, ultimately, to improve existing RBC generation strategies.

## 2 MATERIALS AND METHODS

### 2.1 Samples

Human UCB was acquired from the Umbilical Cord Blood Bank, Beijing, China. Human Runx1C‐H9 cells were provided courtesy of Prof. Dan Kaufman of the University of California (San Diego), San Diego, CA, USA. We applied single cell RNA sequencing on days 21 and 23 of UCB‐E and PSC‐E. All procedures involving human subjects in this study were approved by the Ethics Committee of the AMMS (Approval No.: AF/SC‐08/02.160).

### 2.2 Generation of erythrocyte from UCBMN and UCB‐CD34 ^+^ cells

UCBMN were isolation from samples by SepMate 50 and lymphoprep, according to the manufacturer's instructions. A four‐stage protocol of production of erythrocytes from UCBMNs was modified from Qin et al.^8^ described before. Stage 1 consisted of enrichment from days 0 to 7. The cells were cultured with 1 × 10^6^/mL SFEM II (STEMCELL Technologies, Vancouver, BC, Canada), 100 ng/mL rhSCF (PeproTech, Rocky Hill, NJ, USA), 50 ng/mL rhIL3 (PeproTech), 30 ng/mL rhIL6 (PeproTech), and 10 ng/mL rhTPO (PeproTech). Stage 2 consisted of proliferation from days 8 to 14. The cells were cultured with 5 × 10^5^/mL SFEM II (STEMCELL Technologies), 100 ng/mL rhSCF (PeproTech), 50 ng/mL rhIL3 (PeproTech), and 3 IU/mL rhEPO (PeproTech). Stage 3 consisted of differentiation from days 15 to 21. The cells were cultured in 5 × 10^5^/mL SCGM (CellGenix, Freiburg im Breisgau, Germany), 50 ng/mL rhSCF (PeproTech), 40 ng/mL rhIGF1 (PeproTech), 3 IU/mL rhEPO (PeproTech), 1 × lipid (Gibco; Thermo Fisher Scientific, Waltham, MA, USA), and 200 ng/mL transferrin (Sigma‐Aldrich, Burlington, MA, USA). Stage 4 consisted of maturation from days 21 to 28. The cells were cultured in 5 × 10^5^/mL SCGM (CellGenix), 3 IU/mL rhEPO (PeproTech), 200× P188 (Gibco), and 200 ng/mL transferrin (Sigma‐Aldrich).

UCB‐CD34^+^ cells were isolation and purified by Mitenyi CD34 MicroBead Kit as the manufacturer's instructions. Erythroid differentiation from UCB‐CD34^+^ cells was based on previously reported.^9^

### 2.3 Erythrocyte generation from PSCs

PSCs were co‐cultured with MEF layer cells in DEME/F12 (Gibco), 20% KSR (Gibco) supplemented with GlutaMax (Gibco), NEAA (Gibco), and 10 ng/mL bFGF (PeproTech). The PSCs were dissolved in TrypleSelect (Gibco) after reaching 80% confluence. From day 0 to 14, Spin EB was performed to induce the PSCs to the mesoblastema stage. The haematopoietic stem cells were collected and the progenitor cells emerged from EB.^10^ From day 15 onwards, the PSC‐derived HSPCs were cultured in the same manner as the Stages 3–4 UCBMNs.

### 2.4 scRNA‐Seq

The unsorted cells were processed for library preparation with a Chromium Single Cell 3′ Reagent Kit v. 3 (10× Genomics, Pleasanton, CA, USA). Indexed libraries were pooled and sequenced on a NovaSeq 6000 platform (Illumina, San Diego, CA, USA) using 150‐bp paired‐end reads.

### 2.5 SCENIC analysis

UMI count matrix data obtained by Seurat were used as the input for the SCENIC v. 1.2.4 package in R (R Core Team, Vienna, Austria) to predict changes in the active TFs during erythropoiesis.^11^ The cisTarget Human motif database v. 9 (https://resources.aertslab.org/cistarget/motif2tf/motifs-v9-nr.hgnc-m0.001-o0.0.tbl) comprising 24,453 motifs was used with its default settings to enrich the gene signatures and isolate targets from them based on *cis*‐regulatory cues. The “aucell” positional argument was used to detect regulon enrichment across single cells. The SCENIC v. 1.2.4 package in R loaded the “binary matrix” result and showed activated regulons. A heatmap was plotted with the pheatmap v. 1.0.12 module in R. The “regulon targets info” was loaded into Cytoscape v. 3.7.2 (https://cytoscape.org/download.html) to construct a network of TFs and their target genes.

### 2.6 Image flow

Cells were washed twice by cooled phosphate‐buffered saline (PBS) with 0.1% BSA firstly, then 0.5–1 × 10^7^ cells stained with 1:500 dilution of antibodies CD235a‐FITC, CD68‐APC, CD99‐BV421, and 10 µM 7AAD (BD, Franklin Lakes, NJ, USA). Washed cells were run on the ImageStreamX Amnis (Merck Millipore, Burlington, MA, USA) and analyzed with IDEAS (Merck Millipore).

### 2.7 Immunofluorescence staining

Cultured cells were fixed with 4% paraformaldehyde for 15 min, washed with cooled phosphate‐buffered saline with 0.1% BSA, permeabilized with 0.1% triton‐100, blocked with 10% donkey serum, and then stained with 1:200 dilution of primary antibodies CD235a (abcam, Branford, CT, USA), CD68 (abcam), CD99 (huabio, Hangzhou, Zhejiang, China), and related secondary antibodies. Samples were covered by mounting medium after stained with 10 μM DAPI (Invitrogen, Carlsbad, CA, USA).

### 2.8 Quantification and statistical analysis

All data analysis was performed in R (version 4.0.3).

## 3 RESULTS

### 3.1 Cell composition of terminally differentiated erythroid cells derived from UCBMNs and PSCs

**FIGURE 1:**
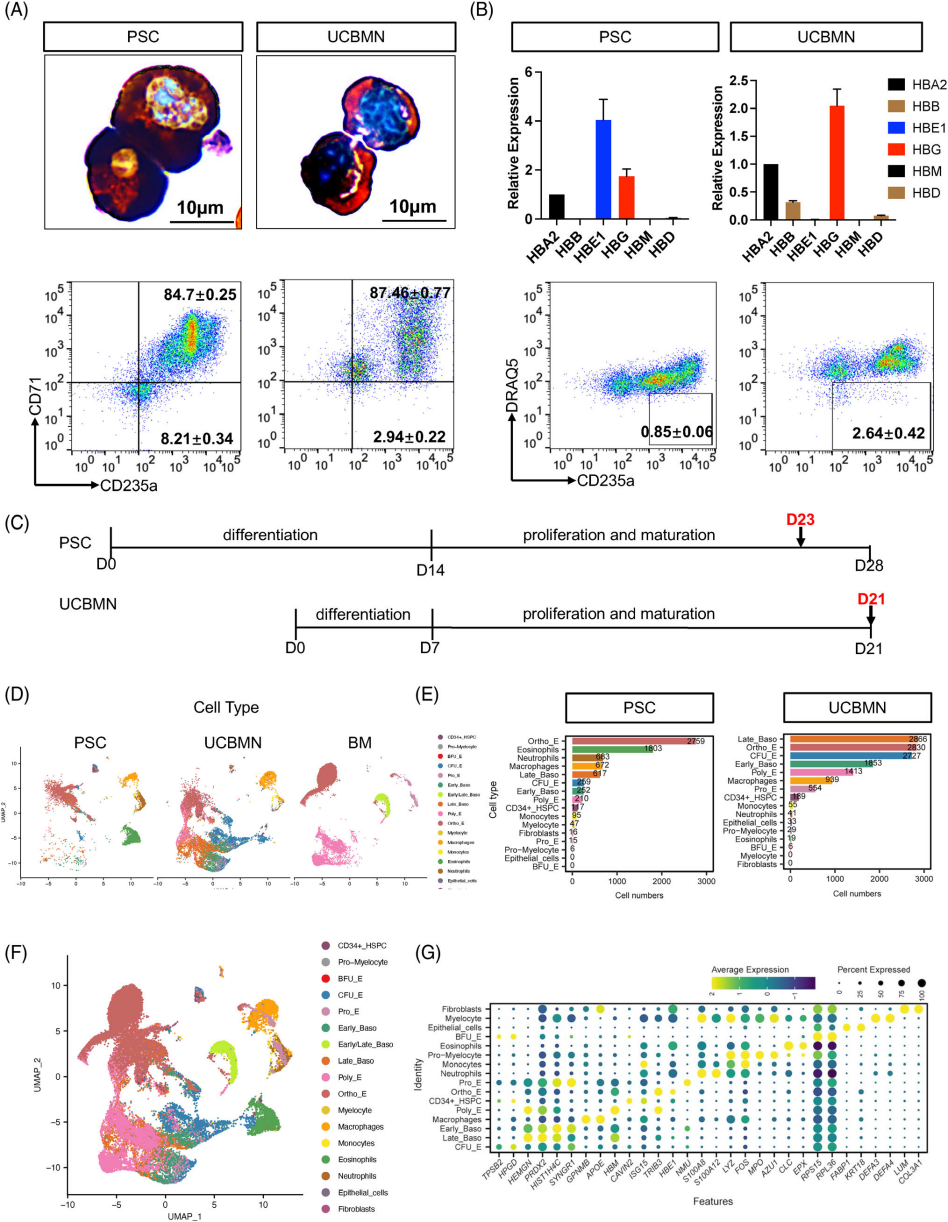
Atlas of pluripotent stem cells (PSC)‐ and umbilical cord blood mononuclear cells (UCBMN)‐derived terminal differentiated cells. (A) Characterization of sequencing samples by Benzidine and Giemsa staining (upper panels) and flow cytometry analysis on CD71/CD235a (lower panels). (B) Comparison of haemoglobin type in sequencing samples by RT–qPCR (upper panels) and flow cytometry analysis on DRAQ5/CD235a (lower panels). Haemoglobin expression were relative to 18S rRNA. The CD235a^+^DRAQ5^−^ cells represent the denucleated cells. (C) Sequencing timepoints of UCBMN‐derived erythropoiesis and PSC‐derived erythropoiesis (PSC‐E) were shown in the schematic diagram. (D) Single cell UMAP plots showing the location of each cell type in samples derived from PSC (left), UCBMN (middle) and BM (right), respectively. (E) Bar plots showing the numbers of each cell type in samples derived from PSC (left) and UCBMN (right), respectively. (F) Single cell UMAP plot of PSC‐E (D23), UCB‐ (D21) and BM sample (GSE150774). Seventeen cell types were indicated by colours. (G) Dot plot showing the expression levels of top two signature genes (ordered by averaged log_2_ fold change, *p* value < 0.05) in each cell type. Colours indicate the scaled average expression of signature genes. Dot sizes indicate the percentage of cells which express the signature genes in each cell type cluster. BFU‐E, burst‐forming unit‐erythroid; CFU‐E, colony‐forming unit‐erythroid; Ortho‐E, orthochromatic erythroblasts; Poly‐E, polychromatic erythroblasts; Pro‐E, pro‐erythroblast.

We applied the spin embryoid bodies and three‐stage differentiation methods to generate mature erythroid cells in vitro (Figure S1A,B). Both cell sources generated erythroid cells with haemoglobin and CD71/CD235a expression (Figure 1A). However, PSCE lacked β‐ or γ‐globin and showed impaired enucleation when compared to UCBE (Figure 1B). We compared UCBE and PSCE at the single‐cell transcriptome level on days 21 and 23 of the experiment, when each reached the highest expression of CD71 and CD235a, to evaluate the differences in their capacity to generate RBCs and to examine the underlying mechanisms for these differences (Figure 1C).

After quality control, 21,105 high‐quality single cells were generated for downstream analysis. The datasets obtained were comparable (Figure S1C). To compare the in vitro terminal erythropoiesis with in vivo erythropoiesis, we further integrated a published dataset from healthy adults which presenting in vivo terminal erythropoiesis (GSE150774).^7^ We annotated 17 reliable cell populations based on R package SingleR and published datasets^9^, ^12^ and the Human Primary Cells Atlas database^13^ (Figure 1D–F; Figure S1D–F). In our own generated data, the cells were classified as progenitors/precursors (e.g., CD34^+^ HSPC, burst‐forming unit‐erythroid [BFU‐E], CFU‐E, and myeloid progenitor cells), erythroid cells (e.g., pro‐erythroblast [Pro‐E], early and late basophilic erythroblasts [Baso‐E], polychromatic erythroblasts [Poly‐E], and Ortho‐E) or non‐erythroid cells (e.g., eosinophils, macrophages, neutrophils, monocytes, myelocytes, epithelial cells, and fibroblasts). BM data convinced our cell annotation in various development stages of erythropoiesis and non‐erythroid cells. As Figure 1D,F showing, compared with PSC, UCBMN present significantly continuous development for their abundant distribution in various erythropoiesis stage cells. The cell compositions of terminally differentiated cells indicated PSC‐ and UCBMN‐derived erythroid cells were nonsynchronous, and the proportional difference for each cell type (erythroid or non‐erythroid cells) between the two systems was clear (Figure 1E). As expected, erythroid cells (91.8%) were dominated in UCBE, while the proportion of erythroid cells (55.9%) was decreased and non‐erythroid cells (44.1%) showed an increased in PSCE. Interestingly, CFU‐Es and macrophages were present in both systems at relatively high percentages, while more non‐erythroid haematopoietic cells (e.g., eosinophils, neutrophils) were present in PSCE but not UCBE (Figure 1E). We calculated the signature gene list of each cluster and showed the top two (ordered by log_2_ fold change, adjusted *p* value < 0.05), these special gene expression patterns were verifiable of the robust cell type annotation results (Figure 1G). Taken together, scRNA‐seq revealed clear differences in the number and diversity of terminally differentiated cells from both PSC‐ and UCBMN‐derived sources.

### 3.2 UCBMN‐ and PSC‐derived terminal erythroid differentiation processes are differentially and dynamically regulated

**FIGURE 2:**
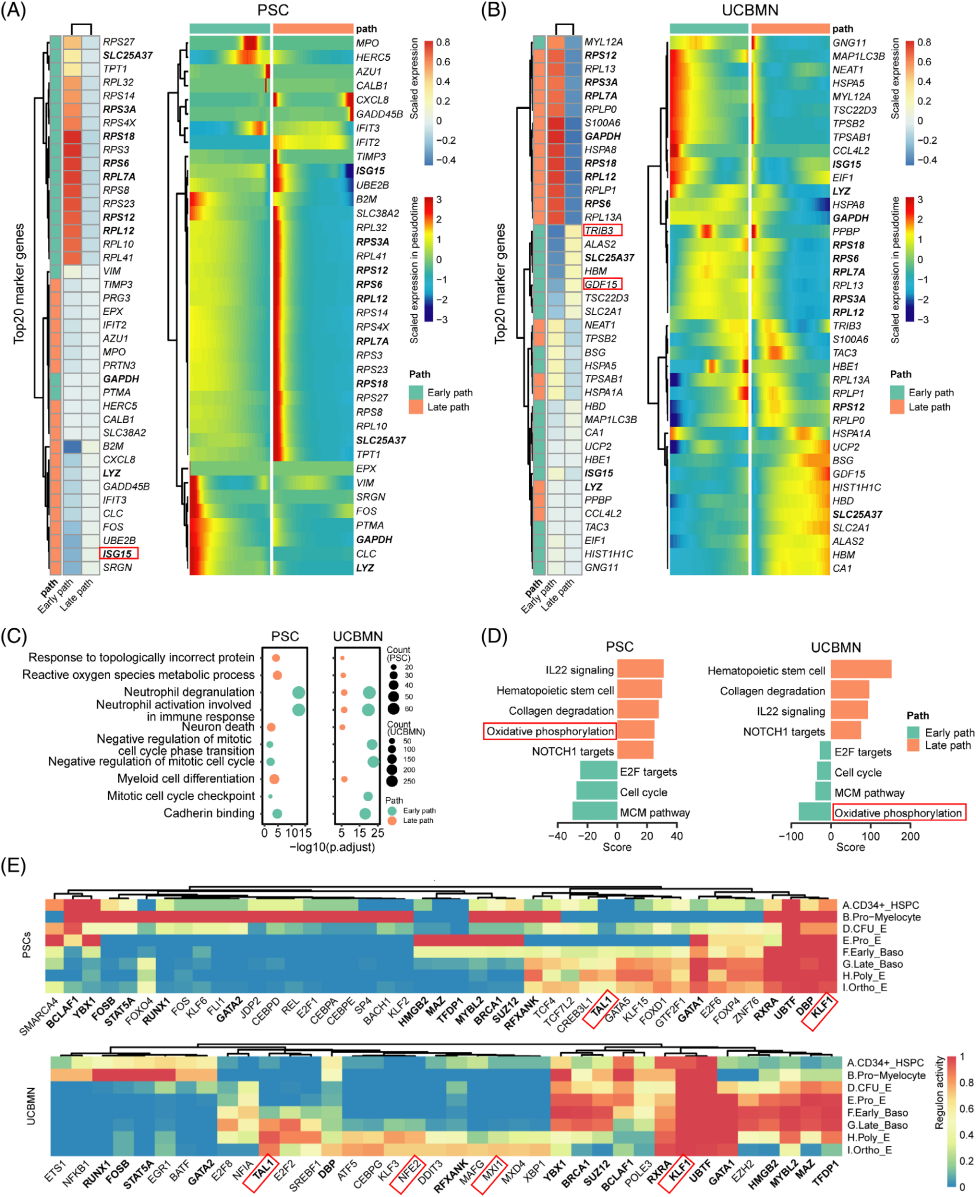
Regulatory dynamics of umbilical cord blood mononuclear cells (UCBMN)‐ and pluripotent stem cells (PSC)‐derived erythropoiesis. (A, B) Heatmap plots are representing the average scaled expression levels (left) and the pseudo‐time expression levels (right) of the top 20 marker genes (ordered by averaged log_2_ fold change, *p* value < 0.05) between the early and late paths in PSC (B) and UCBMN (B) derived progenitors and erythroid cells. (C) Dot plot showing GO annotation of PSC (left) and UCBMN (right) derived cells in the early and late paths. (D) The Bar plot is showing GSVA enrichment scores of PSC (left) and UCBMN (right) derived cells in the early and late paths. (E) Heatmap showing active regulon scores for terminal erythroid cell type from UCBMN and PSC origin. CFU‐E, colony‐forming unit‐erythroid; Ortho‐E, orthochromatic erythroblasts; Poly‐E, polychromatic erythroblasts; Pro‐E, pro‐erythroblast.

Based on the cell compositions of terminally differentiated cells, we observed that PSCE and UCBE were continuous and dynamic. We used Monocle^14^ to construct pseudo‐time trajectories of erythroid differentiation in both processes and clarified the key driving factors of each. The branch point position of the pseudo‐time trajectories indicates that cells from both origins differentiated along distinct early and late paths (Figure 2A,B; Figure S2A–C). Correlation analysis showed that four samples could be divided into early and late paths (Figure S2D). We then compared the differentially expressed genes (DEGs) in the early and late paths of both pseudo‐time trajectories, analysed the expression of the top 20 DEGs of each. We observed conservatively higher expression of ribosomal proteins during the early path compared to that during the late path, indicating ribosome biogenesis is an indispensable, early event for both PSCE and UCBE. We also observed conserved genes, such as solute carrier family 25 member 37 (*SLC25A37*) and interferon‐stimulated gene 15 (*ISG15*), which exhibited an opposite expression pattern in both pseudo‐time trajectories (Figure 2A,B). Slc25a37 was reported to participate in mitochondrial haeme biosynthesis machinery in developing RBCs,^15^ thus *SLC25A37* expression could play a role in haeme biosynthesis, in the late path of UCBE, but not the early path of PSCE.

Using trajectory analysis,^16^ we identified stress erythropoiesis‐related genes, such as Tribbles pseudokinase 3 (*TRIB3*) and growth differentiation factor 15 (*GDF15*),^17^, ^18^ which were expressed in UCBE and highly expressed during the late path. The expression of globin gene haemoglobin subunit epsilon 1 (*HBE1*) occurred earlier in cell differentiation than that of haemoglobin subunit delta (*HBD*) or mu (*HBM*) during UCBE. We detected no globin‐related genes among the top 20 DEGs in PSC‐derived cells (Figure 2C). Meanwhile, in the differentiation trajectory of PSC‐derived erythroid cells, we observed that growth arrest and DNA damage inducible beta (*GADD45B*) were specifically expressed in certain cells in PSCE. Several studies report that *GADD45B* regulates the differentiation of myeloid cells,^19^, ^20^, ^21^ which could be relevant to the higher proportion of myeloid cells in PSC‐derived terminally differentiated cells. Furthermore, we performed gene set variation analysis^22^ and identified several conserved functions in erythroid differentiation, including rRNA processing, IL‐22 signalling, and cell cycle processes (Figure 2D). However, the oxidative phosphorylation pathway was enriched in the early path of UCBMN‐derived cells but not in the late path, while reversed in the PSC‐derived cells, suggesting a dramatic difference in energy demand during ex vivo erythropoiesis from the different cell origins.

Considering the pivotal role of transcription factors on erythropoiesis, we further used SCENIC to illustrate the activation of regulons at different stages of erythropoiesis from both origins.^11^ The dynamic and specific regulation at different development stages throughout erythropoiesis has been demonstrated. The expression of certain transcription factors, such as *RUNX1*, *KLF1*, *TAL1*, and *GATA1*, was conserved during erythropoiesis from both cell origins. Moreover, the differential regulation of transcription factors in terminal erythroid cell types was evident. The regulons, represented by *KLF3*, *NFE2*, and *MXI1*, were specifically presence in UCBMN‐derived Ortho‐Es but not PSC‐derived cells (Figure 2E), implying that specific Ortho‐E regulons and their corresponding gene regulatory networks were related to erythroid maturation.

### 3.3 Comparison of canonical enucleation‐related gene expression in two source‐derived cells

Erythroblasts develop from proerythroblast and then after three or four cell divisions into the Baso‐E, Poly‐E, and Ortho‐E cells that couple with haemoglobin accumulation and chromatin condensation.^23^ Various regulators, comprising epigenetic regulators such as *UBE2O* and *ASXL1*,^24^, ^25^ transcription factors such as *FOXO3*,^26^ and cytoskeletal proteins such as *gelsolin* ^27^ are involved in the enucleation process.^28^

**FIGURE 3:**
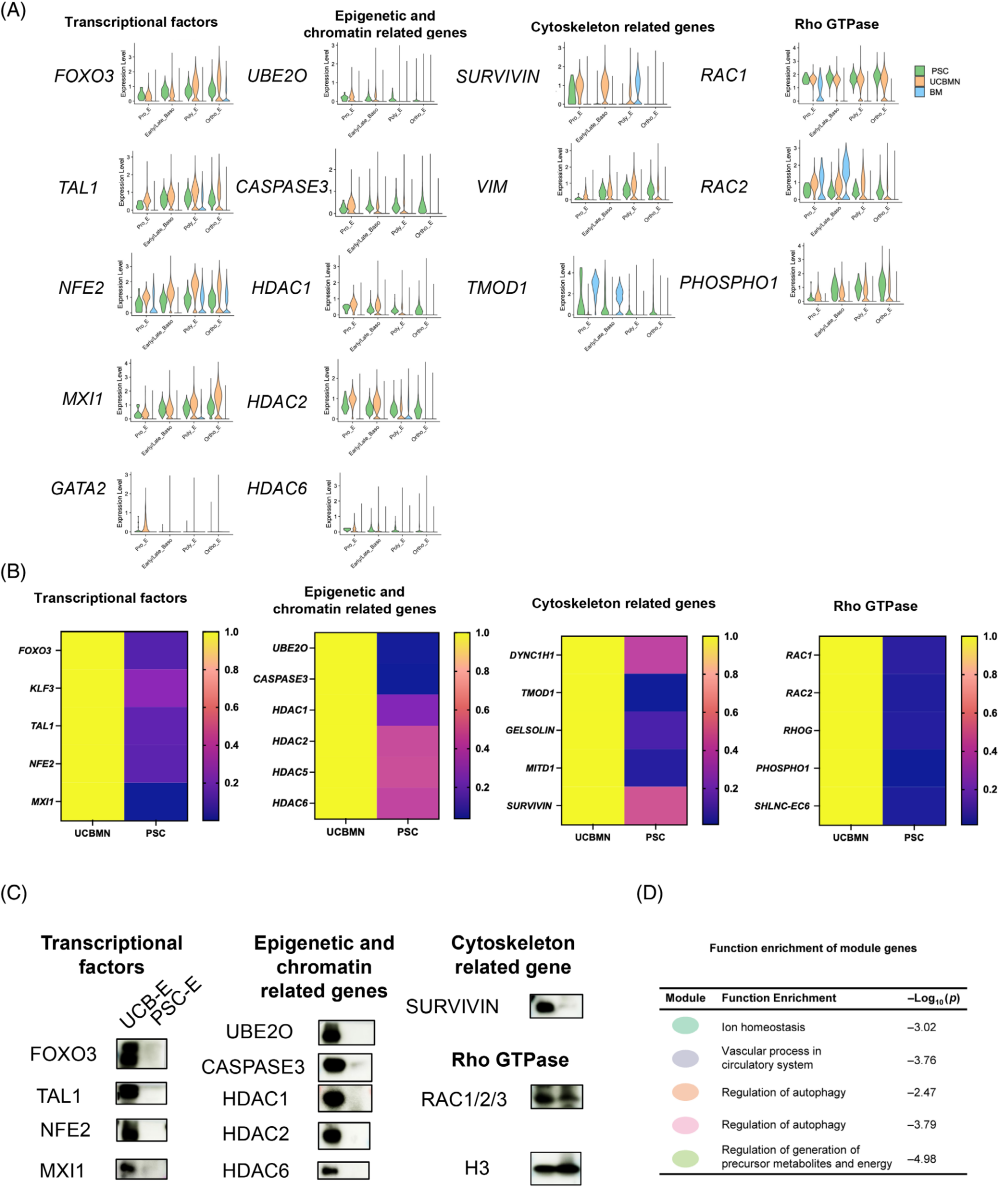
Comparison of canonical enucleation‐related gene expression in two source‐derived cells. (A) Violin plot showing enucleation‐related gene expression in erythroid cells derived from pluripotent stem cells (PSC), umbilical cord blood mononuclear cells (UCBMN) and BM at single cell transcriptional level. (B) Heatmap showing enucleation‐related gene expression in erythroid cells derived from UCBMN and PSC. RT–qPCR was performed on D21 and D28 of UCBMN and PSC. Gene expression was related to 18S rRNA. (C) Western blot assay for enucleation‐related protein in erythroid cells derived from UCBMN (Day 21) and PSC (Day 28) origin. Histone H3 served as a reference for protein expression in different samples. (D) Functional enrichment analysis of enucleation‐related TFs *NFE2*, *KLF3*, *MXI1*, and *TAL1* and their target genes. Ortho‐E, orthochromatic erythroblasts; Poly‐E, polychromatic erythroblasts; Pro‐E, pro‐erythroblast; PSC‐E, PSC‐derived erythropoiesis; UCB‐E, UCBMN‐derived erythropoiesis.

To further illustrate the mechanisms behind the differences in erythroid enucleation between PSCE and UCBE, we compared the transcript expression of canonical enucleation‐related genes at the single cell level. The data showed higher expression of epigenetic and chromatin‐related genes, Rho GTPase genes, and transcriptional factors in the terminally differentiated erythroid cells (especially ortho‐E) from UCBE compared to that in PSCE. Although BM data showed generally low transcript levels for the sample, the in vivo erythropoiesis data basically supports our conclusion (Figure 3A). We further performed RT–qPCR to compare these genes in cells generated from both origins. UCBE showed significantly higher expression of epigenetic‐ and chromatin‐related genes (*UBE2O*, *CASPASE 3, HDAC 1, HDAC 2, HDAC 6*), Rho GTPase genes (*ASXL1, RAC1, RAC3*, and *RHOG*), and erythroid master transcriptional factors (*FOXO3, TAL1*; Figure 3B). Western blotting verified the extremely high expression of the transcription factors (FOXO3, TAL1, NFE2, MXI1) in UCBE that was abolished in PSCE. Moreover, we also verified the higher expression of epigenetic‐ and chromatin‐related proteins and Rho GTPase genes in UCBE compared with that in PSCE (Figure 3C).

Furthermore, to investigate the functions of active regulons in UCBMN‐derived Ortho‐Es, we constructed interacting networks with the functional regulon target gene sets. In contrast to PSCs, we observed highly enriched autophagy, ferroptosis, ion homeostasis, and cellular response to oxygen levels in UCBMN‐derived Ortho‐Es, a stage closely related to erythroid maturation. Autophagy occupied the most target gene modules (Figure 3D; Figure S2E,F). Collectively, the impaired epigenetic regulation, regulons, as well as the downregulation of RhoG GTPase in PSCE could account for the low‐enucleation rate in this process.

### 3.4 Profiling comparison of haemoglobin expression between UCBMN and PSC origin

**FIGURE 4:**
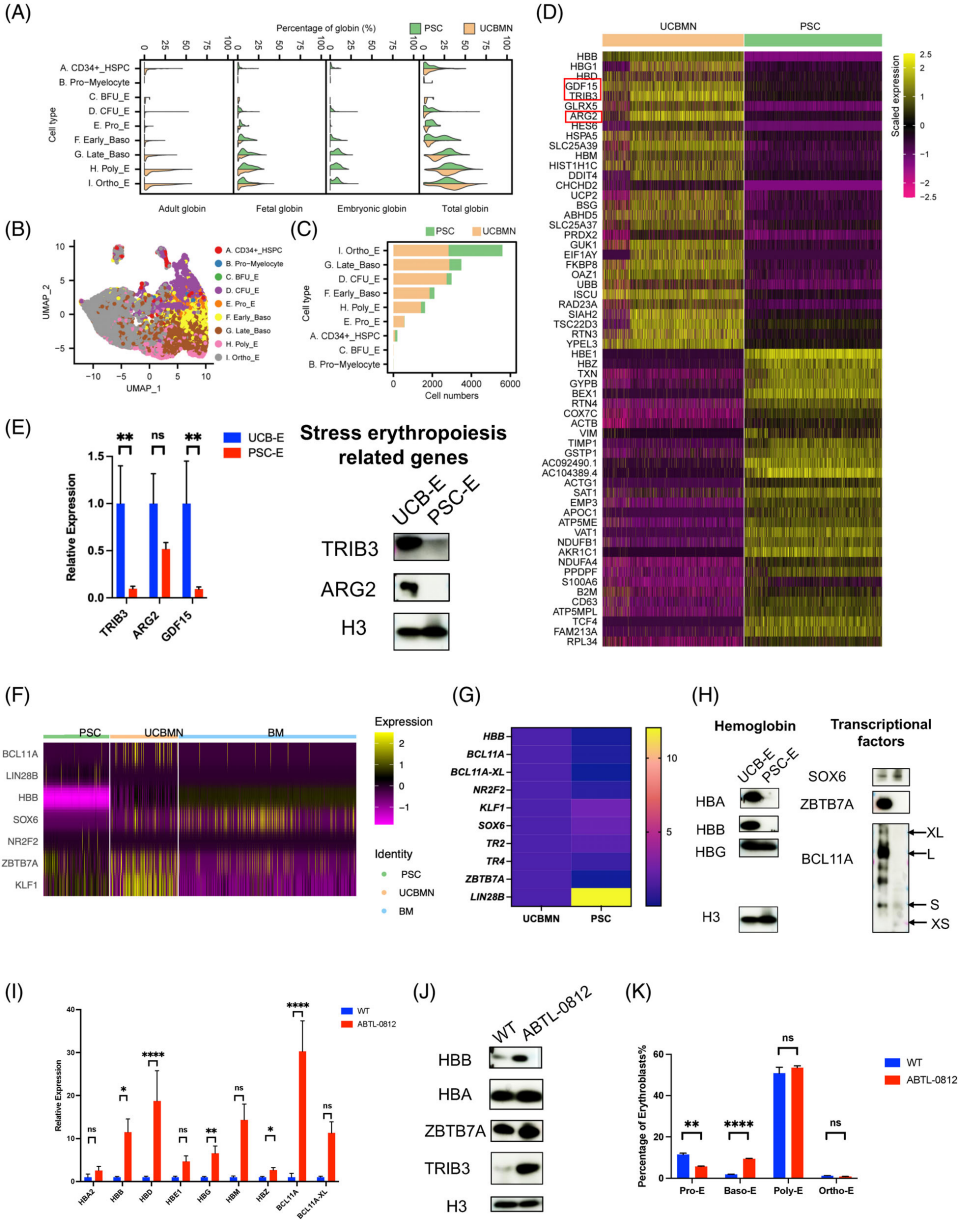
Profiling comparison of haemoglobin expression and the difference in stress erythropoiesis between umbilical cord blood mononuclear cells (UCBMN) and pluripotent stem cells (PSC) origin. (A) Violin plot showing % of adult, fetal, embryonic and total globin for PSC‐derived (green) and UCBMN‐derived (yellow) progenitors and erythroid cells. (B) UMAP atlas of PSC‐ and UCBMN‐derived progenitors and erythroid cells, respectively. Labels indicate cell type annotations performed by SingleR. (C) Bar plot showing numbers of each cell type in A and proportions of cells of UCBMN and PSC origin (different colours). (D) Heatmap showing scaled average expression of top 30 most variable differentially expressed genes (in order of log_2_ FC) in PSC‐ and UCBMN‐derived orthochromatic erythroblasts (Ortho‐E). (E) RT‐qPCR and western blot for the mRNA and protein expressions of stress erythropoiesis related genes. *18S* rRNA and histone H3 served as a standard for gene transcription and protein translational level in different samples. **p < 0.01. (F) Heatmap showing the difference of haemoglobin switching related gene expression between erythroid cells derived from PSC, UCBMN, and BM origin in sc‐RNA level. (G) RT‐qPCR for the mRNA expressions of fetal‐adult globin switch related genes of UCBMN and PSC generated cells at 21 and 28 days (*n* = 3). 18S rRNA served as a standard for gene transcription level in different samples. (H) Western blot assay for HBB, HBA, HBG, and haemoglobin switching related proteins in erythroid cells derived from UCBMN and PSC origin. Histone H3 as a standard for protein expression in different samples. (I) RT‐qPCR for the relative mRNA expressions of hemoglobin and globin switch related genes in control (blue) and ABTL‐0812 (red) treat group. PSC derived erythroid cells were treated with 10 µg/mL TRIB3 agonist ABTL‐0812 from D14 to D28. *p < 0.05, **p < 0.01, ****p < 0.0001. 18S rRNA as a standard for gene transcription level in different samples. (J) Western blot assay for HBB, HBA, ZBTB7A and TRIB3 in control and ABTL‐0812 treated group. Histone H3 served as a standard for protein expression in different samples. (K) The cell population of erythroblast were tested by flow assay in the control and ABTL‐0812 treat group. **p < 0.01, ****p < 0.0001. BFU‐E, burst‐forming unit‐erythroid; CFU‐E, colony‐forming unit‐erythroid; Poly‐E, polychromatic erythroblasts; Pro‐E, pro‐erythroblast; PSC‐E, PSC‐derived erythropoiesis; UCB‐E, UCBMN‐derived erythropoiesis.

Globin expression is a critical issue in erythropoiesis both in vivo and ex vivo. To explore the difference in the expression of different types of globin genes, we compared globin gene expression in 16,702 progenitors and erythroid cells (CD34^+^ HSPCs, myeloid progenitor cells, BFU‐Es, CFU‐Es, Pro‐Es, early Baso‐Es, late Baso‐Es, Poly‐Es, and Ortho‐Es). Generally, the expression of globin genes was gradually upregulated as erythropoiesis progressed. UCBMN‐derived erythroblasts mainly expressed adult‐ and fetal‐type globins, whereas PSC‐derived erythroblasts mainly expressed embryonic‐ and fetal‐type globins (Figure 4A; Figure S3A,B). We compared the numbers of each erythroid cell type and found that Ortho‐E cell numbers were relatively equal between the two systems (Figure 4B,C; Figure S3C). To explore the mechanism behind the difference in globin expression, we compared the DEGs in Ortho‐Es from UCBE and PSCE. Among the top 30 DEGs, we observed that *TRIB3*, *GDF15* and arginase 2 (*ARG2*) were related to stress erythropoiesis and were significantly upregulated in Ortho‐Es, in UCBE compared to that in PSCE (Figure 4D,E; Figure S3D,E), which indicating that the lack of β‐globin in PSCE may be attributable to deficiencies in stress erythropoiesis regulation, such as via TRIB3, ARG2 and GDF15.

Based on the globin gene expression profile, we noticed a globin‐switching block might occur in PSCE. Throughout human ontogeny, the expression of the fetal globin genes would be gradually silenced and replaced with the expression of adult globin genes pre‐ and post‐birth.^29^ Therefore, we further investigated the canonical haemoglobin switching‐related gene expression in the two erythropoietic processes. Clearly, BM demonstrates markedly elevated expression levels of HBB in contrast to both PSC and UCBMN. Additionally, BM exhibits a pronounced correlation with UCBMN in higher expression of beta‐globin suppressors such as BCL11A, alongside decreased expression of the gamma‐globin suppressor Lin28B. Moreover, in comparison to PSC and UCBMN, BM displays notably heightened activity in ZBTB7A and SOX6, while lacking expression of KLF1. These findings strongly imply that the absence of BCL11A, ZBTB7A, and SOX6 expression may serve as pivotal factors contributing to the diminished expression of adult haemoglobin in erythroid cells derived from PSC (Figure 4F), this globin switching dysregulation was also confirmed with RT–qPCR or Western blotting (Figure 4G,H). The protein isoform eXtra‐Long of BCL11A in humans was reported in bone marrow definitive erythropoiesis.^30^ These results suggested impaired haemoglobin switching at both transcription and protein levels during ex vivo PSCE.

To further test whether the lack of β‐globin in PSCE is attributes to the downregulation of stress erythropoiesis‐related genes, we added ABTL‐0812 (10 μg/mL), a TRIB3 agonist, from days 14 to 28 of PSCE. The treatment led to TRIB3 overexpression and significantly increased globin expression, especially adult globin (Figure 4I,J). Furthermore, we observed an increase in Baso‐E cells during terminal erythropoiesis after ABTL‐0812 treatment (Figure 4K). Interestingly, the treatment led to an increase in the expression of γ‐globin repressors (Figure 4I,J). Our results suggest that TRIB3 could increase haemoglobin expression and erythroblast differentiation to facilitating terminal erythropoiesis of PSCE.

### 3.5 CD99^high^ progenitor cells represent a novel subpopulation of cells with higher proliferation ability

**FIGURE 5:**
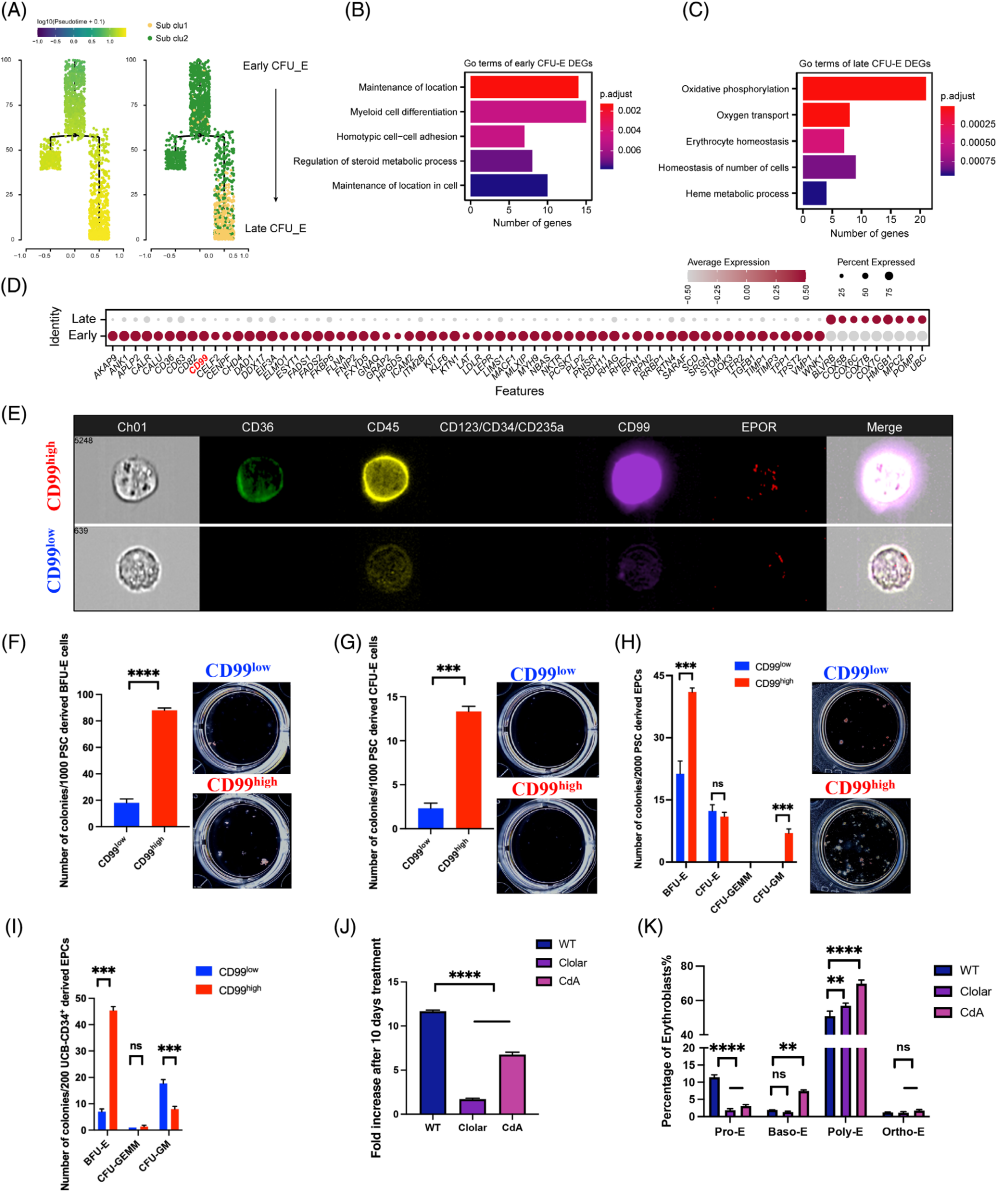
CD99^high^ population generates more erythroid progenitors than the CD99^low^ population. (A) Developmental pseudo‐time trajectory of colony‐forming unit‐erythroid (CFU‐E) cells labelled by pseudo‐time scores (left) and CFU‐E subsets (right). The arrow indicates the direction of differentiation. (B, C) Bar plot representing GO term enrichment results for differentially expressed genes (DEGs) between early (B) and late (C) CFU‐E subsets. Bar colours indicate adjusted *p*‐values of GO terms. (D) Dot plot showing membrane‐encoding DEGs between early and late CFU‐E cells. Colours indicate scaled average gene expression levels. Dot size indicates % of cells expressing genes in early and late CFU‐E subsets. Membrane‐encoding genes were obtained from UniProt under the keywords membrane protein AND organism: “*Homo sapiens* (Human) [9606],” then overlapped the DEGs between early and late CFU‐E cells to achieve the genes list as presented. Candidate marker gene CD99 were marked as red. (E) Image flow analysis of CD99 expression in UCB‐generated CFU‐E (CD235a^−^CD123^−^CD71^+^CD34^−^CD36^+^) cells. The image of cells was captured by Amnis ImageStreamX. (F–H) Bar plot of numbers and representative pictures of colonies formed by pluripotent stem cells (PSC)‐derived burst‐forming unit‐erythroid (BFU‐E) (F) CFU‐E (G) and CD71^+^CD235a^−^ erythroid progenitor cells (EPCs) (H) subpopulations in H4636 medium (*n* = 3). ***p < 0.001, ****p < 0.0001. (I) Bar plot of numbers and representative pictures of colonies formed by UCB‐CD34+ cells derived EPC subpopulations in H4434 medium (*n* = 4). ***p < 0.001. (J) Bar plotting the fold change of erythroblasts after PSC derived cells were treated with antagonist of CD99. PSC derived cells were treated with antagonist of CD99, 0.3 µM Clolar and 0.2 µM 2‐CdA, from D14 (*n* = 3). ****p < 0.0001. (K) Bar plot showing flow cytometry analyzed erythroblast after CD99 antagonist treatment of PSC‐derived cells (CD235a^+^CD49d^hi^Band3^neg^ Pro‐E, CD235a^+^CD49d^hi^Band3^low^ Baso‐E, CD235a^+^CD49d^hi^Band3^med^ Poly‐E, CD235a^+^CD49d^med^ Band3^med^ Ortho‐E) **p < 0.01, ****p < 0.0001. Ortho‐E, orthochromatic erythroblasts; Poly‐E, polychromatic erythroblasts; Pro‐E, pro‐erythroblast.

We are aware that relatively high percentages of CFU‐E cells are present in terminally differentiated cell populations. Our flow cytometry and CFU assay detected a low percentage of CFU‐E during terminal PSCE or UCBE (Figure S4A). We thus compared the transcript expression in developmental trajectories of CFU‐E and HSPC using Monocle. This revealed robust subclusters of new progenitor cells of CFU‐Es (Figure 5A). HSPCs developed into early and late CFU‐E cells along the erythropoiesis path (Figure 5A; Figure S4B–E). A GO enrichment analysis showed that early CFU‐Es were enriched mainly in location maintenance and myeloid differentiation, indicating that early CFU‐Es corresponded to haematopoietic progenitor cells. However, late CFU‐E cells were enriched in oxidative phosphorylation and oxygen transport, which correspond to definitive erythroid progenitor cells (EPCs) (Figure 5B,C; Figure S4F). A cell cycle analysis showed that 15% and 6% of early and late CFU‐E cells, respectively, were in phase S (Figure S4G).

CD99 is a surface glycoprotein related to T cell migration and acute carcinoma progression.^31^, ^32^, ^33^, ^34^ In bone marrow CD34^+^ cells, the CD99^high^ population has a higher migration potential than the CD99^low^ population.^35^ Among the most highly variable genes between the two subclusters, we selected *CD99* to distinguish between early and late progenitor cells (Figure 5D; Figure S4H). CFU‐E subpopulations were isolated from UCBMN‐derived cells as previously reported^12^ (Figure S4M). Giemsa staining revealed that CD99^high^ cells had larger nuclei than CD99^low^ cells (Figure S4I). The larger nuclei occurred mainly among the early progenitor cells. Flow cytometry imaging and FACS analysis of the haematopoietic progenitor cells revealed that CD99 expression levels in the CFU‐E cells were consistent with the results of the transcriptome analysis (Figure 5E; Figure S4H).

By integrating the human bone marrow data,^6^ we confirmed that early and late CFU‐E cells in vivo were also marked by CD99^high^ and CD99^low^ expression (Figure S5A–D). The GO terms showing DEGs enriched in metabolic pathways indicate that the CD99^high^ subpopulation was enriched in haematopoietic progenitor cell differentiation‐related haemopoiesis regulation, whereas the CD99^low^ subpopulation was enriched in erythrocyte maturation‐associated chromosome condensation (Figure S5E). Thus, the CD99^high^ subcluster was predicted to have high in vivo proliferation capacity. We isolated the CD99^high^ subpopulation from PSC‐derived BFU‐E, CFU‐E and CD71^+^CD235a^−^ EPCs, which generated larger and more numerous colonies than the CD99^low^ subpopulation (Figure 5F–H). We verified these trends with UCB‐CD34^+^ cell‐derived CD71^+^CD235a^−^ EPCs using the CFU assay (Figure 5I). The CD99^high^ subpopulation in PSC and UCB‐CD34^+^ cell‐derived EPCs both formed granulocyte–macrophage progenitors while BFU‐Es were dominant in the CD99^low^ subpopulation, suggesting that the CD99^high^ subcluster retains the potential of granulocyte, macrophage, and erythroid cell differentiation, further suggesting that the CD99^high^ subpopulation presents these progenitor cells at an early stage. When we added the CD99 antagonists clofarabine and 2‐chlorodeoxyadenosine from day 14 to 28 of PSCE, cell expansion was decreased (Figure 5J). More importantly, we observed increased production of Baso‐E and Poly‐E cells and decreased Pro‐E cells by flow cytometry (Figure 5K), which suggests that CD99 not only marked the proliferation subpopulation but also was involved in the maturation of erythroid cells, implying that CD99 antagonists can be used to promote erythroid maturation in vitro.

### 3.6 Macrophages were involved in ex vivo erythropoiesis by cell–cell contact

**FIGURE 6:**
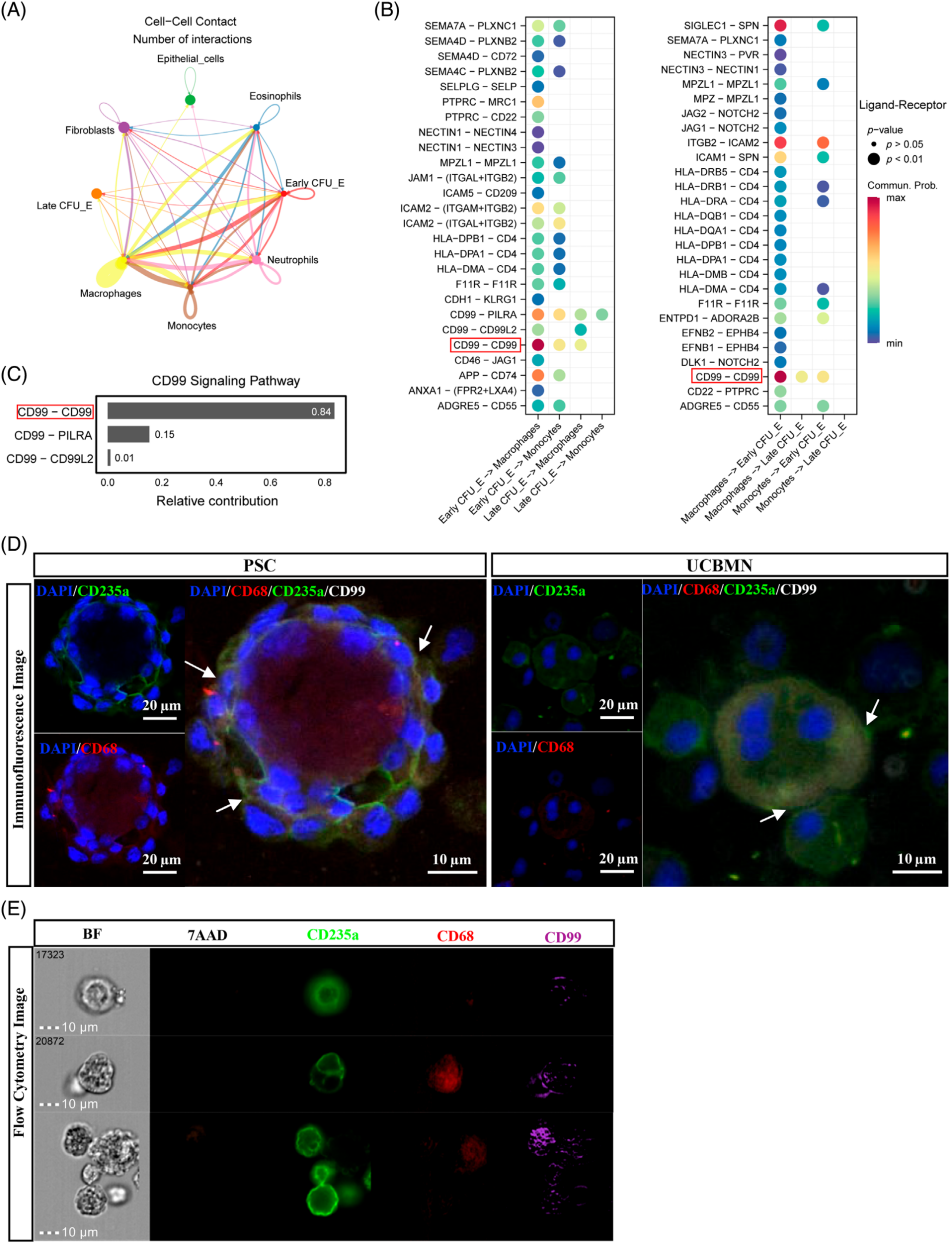
Macrophage contacts erythroid cells during terminal erythropoiesis ex vivo. (A) The cell–cell communication network showing the number of interactions between two cell groups at cell–cell contact pattern. (B) The dot plot showing the significant ligand‐receptor pairs from the early and the late colony‐forming unit‐erythroid (CFU‐E) cells to macrophages and monocytes (left), and from macrophages and monocytes to the early and the late CFU‐E cell at cell–cell contact pattern (right). Dot size represents the statistical significance of ligand‐receptor pairs, and colour represents the communication probability of ligand‐receptor pairs. (C) The bar plot showing the relative contribution of each ligand‐receptor pair to the overall CD99 signalling pathway. (D) Representative immunofluorescence images showing expression of CD99 in pluripotent stem cells (PSC) and umbilical cord blood mononuclear cells (UCBMN)‐derived macrophage and erythroid cells. PSC‐derived cells on D23 and UCBMN‐derived cells on D21 were fixed and stained as indicated in the methods section. Images were captured by using 20× and fluorescent filters optimized for observing DAPI‐stained nuclei (blue), 647‐labelled CD99 (white), 488‐labelled CD235a (green) and 568‐labelled CD68 (red). White arrows indicate CD99 expression on the contact area between macrophages and erythroid cells. Images obtained from the same field with different filters were merged. Scale bar = 10 μm. (E) Image flow analysis of CD99 expression on PSC‐derived macrophage and erythroid cells. Image of cells was captured by using 20× and fluorescent filters of Amnis ImageStreamX for observing 7AAD‐stained nuclei (super red), BV421‐labelled CD99 (purple), FITC‐labelled CD235a (green) and APC‐labelled CD68 (red). Scale bar = 10 μm.

As illustrated in Figure 1A, ca. 10% of cells were double‐negative for CD71/CD235a after terminal erythroid differentiation. The most significant non‐erythroid cells that were in contact with erythroid cells were macrophages. CFU‐Es in our data were divided into early and late CFU‐E cells with different levels of CD99 expression. Macrophage–CFU‐E cell communication decreased as CFU‐Es developed (Figure 6A). We used cell–cell contact and cell signalling databases in CellChat (version 1.1.2) of R to predict the ligand–receptor interactions between macrophages and CFU‐E cells. We identified a total of 30 and 36 ligand‐receptor interaction pairs from macrophages to erythroid cells and erythroid cells to macrophages, respectively. Communication from macrophages to erythroid cells was more significant than the opposite (Figure 6B). Furthermore, CD99–CD99 was the most significant ligand–receptor pair (Figure 6B,C). To examine the contact of macrophage and erythroid cells by CD99–CD99, immunofluorescence staining and flow cytometry analysis of CD99 in the two cell types were performed. This revealed CD68^+^ macrophage contacts with CD235a^+^ erythroid cells and CD99 expression in the contacting area (Figure 6D,E). Our results demonstrated that macrophages were involved in ex vivo erythropoiesis based on *CD99* expression level, and specialized erythroid cells interacted with macrophages by establishing a CD99–CD99 contact, which is a novel mechanism through which macrophages participate in erythropoiesis.

## 4 DISCUSSION

To clarify the mechanisms underlying the known limitations of RBC maturation and production ex vivo, we decoded the single‐cell transcriptomics of UCBMN‐ and PSC‐derived terminal erythroid cells. At the sequencing timepoint, morphological analysis of cells generated from the two sources showed that the cells reached the terminal stage of erythropoiesis (Figure 1A). Appropriately 90% of cells collected from both sources entered erythropoiesis, while 10% of the cells remained both CD71^−^ and CD235a^−^, including non‐erythroid cells and quiescent progenitors, which has not been previously described. Some progenitors, such as CD34^+^, BFU‐E and CFU‐E cells, remained in an undifferentiated state, which might be due to the heterogeneity, epigenetic modifications, or metabolic programming of the origin cells. We noted the diversity in the terminally differentiated cells, and the cell populations originating from the two erythropoietic sources were distributed in various developmental stages, consistent with the continuous differentiation model. Cell–cell interaction analysis suggests that besides macrophages, other non‐erythroid cells, including myelocytes, monocytes, neutrophils and fibroblasts could also be involved in and facilitate in vitro erythropoiesis.

Macrophages participate in erythroid differentiation as a microenvironment component and contact erythroid cells via specific protein binding.^36^, ^37^ We observed marked differences in macrophage proportions in terminally differentiated cells from both sources, suggesting that macrophages were distinct, probably differentiated unequally and performed different roles for each source. Besides erythroid cells, macrophages also facilitate the expansion of haematopoietic cells. Macrophage have been reported to be involved in the proliferation of EPCs and enucleation of erythroid cells.^38^, ^39^, ^40^, ^41^, ^42^ Gao et al. reported that macrophages facilitate the formation of functional haematopoietic stem cell/multipotent progenitor units in the fetal liver to promote haematopoietic stem cell expansion through growth factor secretion.^43^ In terms of proliferation, CFU‐E progenitors undergo four or five terminal cell divisions then giving rise to erythroblasts,^44^ and the CD99^low^ subcluster could present the terminal of the proliferation period. Our data suggest that CD99 could regulate the proliferation of erythroid progenitors, involving macrophage communication with CFU‐E erythroid progenitors through CD99–CD99 signalling. Interestingly, a decreased expression of CD99 during erythroid differentiation was observed and we believe that the disappearance of CD99 may be necessary for terminal erythropoiesis (Figure S6A,B); during this time, the nuclear phagocytosis of Ortho‐E cells is performed by macrophages and reticulocytes are released from macrophages with down‐regulated CD99 expression (Figure S6C). Collectively, this study provided a mechanism for the role of macrophages in erythropoiesis.

We observed that stress erythropoiesis specifically occurred in UCBMN‐derived ortho‐Es, and related genes including *TRIB3*, *ARG2*, and *GDF15* were upregulated. *TRIB3* strongly responded to insufficient erythropoietin in adult bone marrow EPCs.^45^ *TRIB* ^−/−^ mice have dropped RBC counts and haemoglobin content. These studies demonstrate that *TRIB3* regulates stress erythropoiesis.^18^ The current study identified the small molecule ABTL‐0812, α‐hydroxylinoleic acid, as an agent that increases haemoglobin expression and promotes erythroid maturation. *GDF15* regulates progenitor metabolism and promotes stress erythropoiesis in mouse models.^17^ *ARG2* confers immunosuppressive properties and is associated with the development of the neonatal immune system^46^ and remains to be evaluated in ex vivo erythropoiesis. In humans, the level of CD71^+^ EPCs was increased in the spleen when stress erythropoiesis occurred.^47^ Culturing HSPCs in a medium containing stem cell growth factor, erythropoietin, and dexamethasone mimics stress erythropoiesis.^48^, ^49^ We hypothesized that UCB‐derived progenitors could resemble stress erythroid progenitors, and UCBE‐enriched transcripts for *TRIB3*, *ARG2*, and *GDF15* were potential regulators of haemoglobin expression. We showed that there were fewer immune cells, including eosinophils and neutrophils, in UCBE compared to that PSCE, which could be related to stress erythropoiesis. A further understanding of the mechanisms behind the in vitro stress erythropoiesis will aid erythrocyte generation.

To the best of our knowledge, this is the first study to identify CD99 as a marker of early and late CFU‐E in UCBE and PSCE, as well as in bone marrow. Furthermore, the CD99^high^ subpopulation in CFU‐E cells was a subcluster with high‐proliferative capacity both in vitro and in vivo. Identification of this subpopulation in the exponential growth period of erythropoiesis, from BFU‐E to CFU‐E cells, provides new insights into the mechanism of erythroid progenitor proliferation. It has been shown that CD99 is involved in cell expansion through the HIF1α–CD99–ERK1/2 pathway.^33^ In this study, we evaluated CD99 expression and observed that it was expressed in the early stages of erythropoiesis and decreased gradually during the process. Reducing CD99 expression using antagonists facilitated erythroid maturation, and could shrink the population of non‐erythroid cells in the culture system. This study provides new knowledge on the role of CD99 in erythroid progenitor proliferation and maturation during ex vivo erythropoiesis.

In this study, we comprehensively compared UCB‐ and PSC‐derived terminally differentiated erythroid cells at the single cell level and elucidated the mechanisms underlying current limitations of in vitro erythropoiesis such as β‐globin expression and enucleation. For the first time, we deciphered the cell composition and differentiating path and determined the regulons involved in regulating terminal erythropoiesis at the single cell level. We observed the heterogeneity of the CFU‐E population that was divided by CD99 expression. We also identified a novel subpopulation with high‐proliferation capacity in erythroid progenitors and its putative role in ex vivo RBC generation, as well as small molecules which increase haemoglobin expression that can be applied to future RBC generation. Future research should endeavour to understand the specific effects of these regulators on erythropoiesis and determine how they can be applied to improve RBC production. These investigations will serve as a reference for potential large‐scale, in‐depth research on RBC generation.

## AUTHOR CONTRIBUTIONS

XW and WZ performed research and analysed the data. XW collected, and interpreted data with the help of HY, TC, RZ, LZ, and XL. WZ performed bioinformation analysis with help of SZ and ZX. XP, XF, ZZ, WY, JX, XW, and WZ designed research, and wrote the manuscript. XP, XF, WY, and ZZ supervised and funded the project. All authors have read and approved the final manuscript.

## FUNDING INFORMATION

This research was supported by the Strategic Priority Research Program of the Chinese Academy of Sciences (No. XDA16010602 to XF), National Natural Science Foundation of China (No. 81870097 to ZZ, Nos. 82070114 and 82270126 to XF, No. 32200589 to MQ, No. 82200690 to LZ and No. 32300612 to RZ), the National Key Research and Development Program of China (Nos. 2017YFA0103100, 2017YFA0103103, and 2017YFA0103104 to XP, No. 2022YFC2406803 to ZZ), and Science and Technology Program of Guangzhou, China (No. 202002030025).

## CONFLICT OF INTEREST STATEMENT

The authors declare no competing financial interests.

## Supporting information


**Data S1.** Supporting information.

## Data Availability

The raw sequence data reported in this paper have been deposited in the Genome Sequence Archive in National Genomics Data Center, China National Center for Bioinfomation / Beijing Institute of Genomics, Chinese Academy of Sciences (GSA‐Human: HRA002190) that are publicly accessible at https://ngdc.cncb.ac.cn/gsa-human/browse/HRA002190. [Correction added on 08 April 2024, after first online publication: The Data Availability Statement has been modified. The original read, “The scRNA‐seq datasets were available in the Genome Sequence Archive for Human (https://ngdc.cncb.ac.cn/gsa-human/s/AdGSYf73). This article does not report the original code. Any additional information required to reanalyze the data reported in this article is available from the lead contact upon request.”.]
